# The prediction of in-hospital mortality in chronic kidney disease patients with coronary artery disease using machine learning models

**DOI:** 10.1186/s40001-023-00995-x

**Published:** 2023-01-18

**Authors:** Zixiang Ye, Shuoyan An, Yanxiang Gao, Enmin Xie, Xuecheng Zhao, Ziyu Guo, Yike Li, Nan Shen, Jingyi Ren, Jingang Zheng

**Affiliations:** 1 Department of Cardiology, Peking University China-Japan Friendship School of Clinical Medicine, Beijing, 100029 China; 2 Department of Cardiology, China-Japan Friendship Hospital, 2 Yinghua Dongjie, Chaoyang District, Beijing, 100029 China; 3 Graduate School of Peking Union Medical College, Chinese Academy of Medical Sciences and Peking Union Medical College, Beijing, 100029 China

**Keywords:** MIMIC-IV database, In-hospital mortality, Chronic kidney disease, Coronary artery disease, Machine learning

## Abstract

**Objective:**

Chronic kidney disease (CKD) patients with coronary artery disease (CAD) in the intensive care unit (ICU) have higher in-hospital mortality and poorer prognosis than patients with either single condition. The objective of this study is to develop a novel model that can predict the in-hospital mortality of that kind of patient in the ICU using machine learning methods.

**Methods:**

Data of CKD patients with CAD were extracted from the Medical Information Mart for Intensive Care IV (MIMIC-IV) database. Boruta algorithm was conducted for the feature selection process. Eight machine learning algorithms, such as logistic regression (LR), random forest (RF), Decision Tree, K-nearest neighbors (KNN), Gradient Boosting Decision Tree Machine (GBDT), Support Vector Machine (SVM), Neural Network (NN), and Extreme Gradient Boosting (XGBoost), were conducted to construct the predictive model for in-hospital mortality and performance was evaluated by average precision (AP) and area under the receiver operating characteristic curve (AUC). Shapley Additive Explanations (SHAP) algorithm was applied to explain the model visually. Moreover, data from the Telehealth Intensive Care Unit Collaborative Research Database (eICU-CRD) were acquired as an external validation set.

**Results:**

3590 and 1657 CKD patients with CAD were acquired from MIMIC-IV and eICU-CRD databases, respectively. A total of 78 variables were selected for the machine learning model development process. Comparatively, GBDT had the highest predictive performance according to the results of AUC (0.946) and AP (0.778). The SHAP method reveals the top 20 factors based on the importance ranking. In addition, GBDT had good predictive value and a certain degree of clinical value in the external validation according to the AUC (0.865), AP (0.672), decision curve analysis, and calibration curve.

**Conclusion:**

Machine learning algorithms, especially GBDT, can be reliable tools for accurately predicting the in-hospital mortality risk for CKD patients with CAD in the ICU. This contributed to providing optimal resource allocation and reducing in-hospital mortality by tailoring precise management and implementation of early interventions.

**Supplementary Information:**

The online version contains supplementary material available at 10.1186/s40001-023-00995-x.

## Introduction

In the past few decades, chronic kidney disease (CKD) has become increasingly prevalent among various countries and regions around the world, increasing the enormous financial burden of many countries [1]. A major cause of death among patients with chronic kidney disease is cardiovascular disease [2], and CKD patients with coronary artery disease (CAD) have a poorer prognosis than CKD patients without CAD [3, 4]. Moreover, the risk factors of patients with CKD combined with CAD are much different from those with only CAD [5]. Some studies demonstrated that atherosclerosis is the leading cause of death in advanced CKD patients with CAD, especially end-stage renal disease (ESRD) patients [6]. In addition, the pathogenesis of CKD patients with CAD has not been clearly elucidated [7]. Thus, the present indicators and prediction models perform poorly in predicting clinical outcomes for CKD patients with CAD.

Machine learning (ML) is a cutting-edge technology with the rapid development of artificial intelligence [8]. Compared to the traditional statistical method, ML has better clinical predictive accuracy and performance with faster processing speed [9]. With the development of the online public standard database, such as the Medical Information Mart for Intensive Care IV (MIMIC-IV), ML has increasingly penetrated the medical analysis field [10]. However, a few ML algorithms focused on the mortality prediction of CKD patients with CAD.

The purpose of our study is to (1) construct novel predictive models based on the various machine learning algorithm for in-hospital mortality of patients with CAD and CKD in intensive care units (ICU); (2) select an ML model with the best predictive performance and clinical value; and (3) validate these ML models via external set from the Telehealth Intensive Care Unit Collaborative Research Database (eICU-CRD) database.

## Methods

### Data sources

Data from the MIMIC-IV database were used in this study to establish predictive models for patients with CKD and CAD [11]. MIMIC-IV was a free, online accessible public database containing more than 50,000 ICU admissions from 2008 to 2019 in Beth Israel Deaconess Medical Center (Boston, Massachusetts). Data from eICU-CRD were used as an external validation cohort [12]. Over 200,000 ICU admissions from 208 hospitals across the country were compiled in the eICU-CRD, which was a publicly available multicenter database. The MIMIC-IV and the eICU-CRD database included the following information: demographics, vital signs, laboratory results, and diagnosis of International Classification of Diseases and Ninth Revision (ICD-9) codes. One author (ASY) obtained the certification to access these databases and extracted variables needed in the study (certification number: 39674606). Patients in these databases were unidentified with their health information, so individual patient consent was not required.

### Study population and data extraction

All patients diagnosed with CAD and CKD were included in this study. Patients who stayed in ICU for less than 6 h, less than 18 years old, without baseline creatinine results, and with missing data > 30% were excluded. Only the first admission was taken into account if a patient had multiple admissions. Baseline creatinine was defined as the creatinine level in the patient's first blood test after hospital admission. Data of demographic information, lab results, hourly vital signs, comorbidities, medications (including aspirin, clopidogrel, ticagrelor, statin, beta-blocker, NOAC, and warfarin), operative procedures, ICU stay details, and in-hospital mortality were extracted from MIMIC-IV and eICU-CRD database using pgAdmin PostgreSQL tools (version 1.22.1).

### Data preprocessing and feature selection

Variables with > 30% missing values were dropped, and multiple imputations were conducted for other vacant data. Multivariate Imputation by Chained Equations (MICE) was performed and returned an object containing five complete datasets. Then, statistical models such as linear regression or generalized linear model were applied to each complete dataset in turn for interpolation modeling. The pool function consolidates these individual analysis results into a group. The complete dataset is finally returned based on the standard errors and P-values of the model. MIMIC-IV and eICU (external validation data) databases were imputed separately using the fully conditional specification to avoid data leakage via the “mice” package in R [13].

Feature selection was a crucial process of reducing the number of features in a massive dataset according to the importance of the study variables. The Boruta algorithm was a wrapper method for feature selection built around the Random Forest Classifier algorithm. During the model construction, Boruta created a copy of the original dataset features as Shadow Features and compared the *Z*-score between the actual features and shadow features calculated via Random Forest Classifier in each iteration. If the *Z*-score of an actual feature was higher than the maximum *Z*-score of shadow features, this feature was considered pivotal and kept; otherwise, it was dropped [14].

### Statistical analysis

Patients were divided into two groups according to whether they survived to discharge. Categorical variables were summarized as numbers with percentages and compared by Fisher’s exact probability method (or Chi-square tests). The Wilcoxon rank sum test was used to test continuous variables that were expressed as the median with interquartile ranges.

Eight machine learning models, including logistic regression (LR), random forest (RF), Decision Tree, K-nearest neighbors (KNN), Gradient Boosting Decision Tree Machine (GBDT), Support Vector Machine (SVM), Neural Network (NN), and Extreme Gradient Boosting (XGBoost), were established to develop the predictive models. 70% of the patients from MIMIC-IV were randomly extracted as the training set, while the remaining 30% was utilized for internal validation. Tenfold cross-validation was performed in each model to prevent overfitting to acquire average accuracy. The performance of each model was evaluated by the area under the receiver operating characteristic (ROC) curve (AUC) and average precision (AP) from precision/recall (P-R) curves in the validation set. Further, the model with the best performance was picked up to recognize the risk factors most related to in-hospital deaths interpreted by Shapley Additive Explanations (SHAP) method. The SHAP value visually exhibited each feature's importance and contribution to in-hospital mortality. In addition, data from eICU-CRD were used as external validation to assess the prediction model's performance. Decision curve analysis (DCA), AUC, and calibration curves were conducted to evaluate the clinical application and the consistency of the predictive probabilities.

All statistical analyses, machine learning algorithms, and SHAP were implemented via Python (version 3.9.12). The Boruta algorithms were conducted by R (version 4.1.3, Austria). A *P*-value lower than 0.05 (two-sided) was regarded as statistically significant.

## Results

### Baseline characteristics

**Fig. 1:**
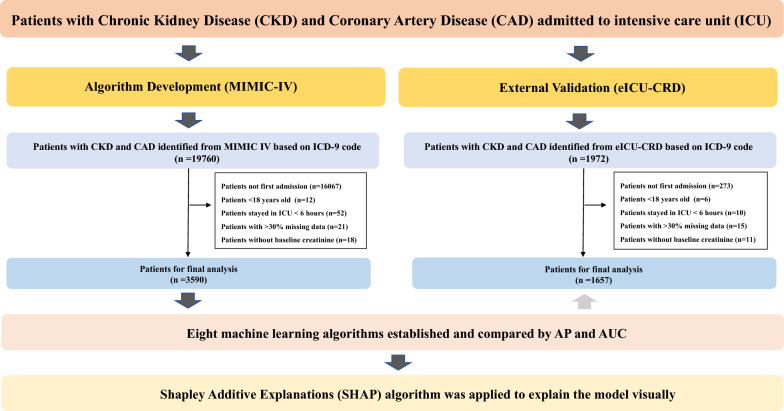
Flowchart of patient selection from MIMIC-IV and eICU-CRD database. *MIMIC* Medical Information Mort for Intensive Care, *eICU-CRD* Telehealth Intensive Care Unit Collaborative Research Database

A total of 3590 CKD patients with CAD from MIMIC-IV and 1657 CKD patients with CAD from eICU-CRD were included in this study cohort according to the inclusion and exclusion criteria. Figure 1 exhibits the screening process. In the MIMIC-IV database, 536 of 3590 (14.9%) CKD patients with CAD died during hospitalization, while 3054 participants survived. The differences in baseline characteristics are summarized in Tables 1, 2. Patients who died during the hospitalization have higher serum creatinine and troponin level and higher myocardial infarction, heart failure, and arrhythmia risks (*P* < 0.001).

**Table 1:** Baseline characteristics, vital signs, laboratory results of patients with CKD and CAD from MIMIC-IV database

	Overall	Survivor	*P*-Value
	3590	3054	
Age (years)	76.0 [68.0, 84.0]	75.0 [68.0, 83.0]	< 0.001
Male, *n* (%)	2451 (68.3)	2100 (68.8)	0.146
los_icu (day)	2.2 [1.2, 4.1]	2.2 [1.2, 4.0]	0.01
scr_baseline (mg/dL)	1.4 [1.1, 2.0]	1.4 [1.1, 1.9]	< 0.001
eGFR (mL/min/1.73 m^2^)	47.2 (24.2)	48.4 (23.8)	< 0.001
CKD stage, *n* (%)			
1	166 (4.6)	142 (4.6)	< 0.001
2	871 (24.3)	792 (25.9)	
3	1581 (44.0)	1373 (45.0)	
4	655 (18.2)	506 (16.6)	
5	137 (3.8)	92 (3.0)	
Dialysis	180 (5.0)	149 (4.9)	
ACS, *n* (%)	1049 (29.2)	886 (29.0)	0.545
Myocardial infarct, *n* (%)	2423 (67.5)	2014 (65.9)	< 0.001
Congestive heart failure, *n* (%)	2313 (64.4)	1925 (63.0)	< 0.001
Peripheral vascular disease, *n* (%)	847 (23.6)	713 (23.3)	0.437
Cerebrovascular disease, *n* (%)	573 (16.0)	468 (15.3)	0.015
Dementia, *n* (%)	189 (5.3)	150 (4.9)	0.031
Chronic pulmonary disease, *n* (%)	1067 (29.7)	890 (29.1)	0.078
Rheumatic disease, *n* (%)	138 (3.8)	119 (3.9)	0.788
Peptic ulcer disease, *n* (%)	111 (3.1)	92 (3.0)	0.602
Diabetes with control, *n* (%)	1359 (37.9)	1181 (38.7)	0.018
Diabetes without_control, *n* (%)	1065 (29.7)	887 (29.0)	0.058
Malignant cancer, *n* (%)	338 (9.4)	265 (8.7)	< 0.001
Mild liver disease, *n* (%)	262 (7.3)	189 (6.2)	< 0.001
Severe liver disease, *n* (%)	74 (2.1)	46 (1.5)	< 0.001
HT, *n* (%)	3300 (91.9)	2811 (92.0)	0.582
Atrial fibrillation or flutter, *n* (%)	1638 (45.6)	1339 (43.8)	< 0.001
Ventricular arrhythmia, *n* (%)	195 (5.4)	129 (4.2)	< 0.001
Cardiac arrest, *n* (%)	160 (4.5)	79 (2.6)	< 0.001
PCI, *n* (%)	195 (5.4)	175 (5.7)	0.075
CABG, *n* (%)	624 (17.4)	610 (20.0)	< 0.001
Aspirin, *n* (%)	3009 (83.8)	2622 (85.9)	< 0.001
Clopidogrel, *n* (%)	1114 (31.0)	975 (31.9)	0.007
Ticagrelor, *n* (%)	2 (0.1)	1 (0.0)	0.276
Statin, *n* (%)	3005 (83.7)	2647 (86.7)	< 0.001
Beta_blocker, *n* (%)	2618 (72.9)	2334 (76.4)	< 0.001
NOAC, *n* (%)	262 (7.3)	243 (8.0)	< 0.001
Warfarin, *n* (%)	908 (25.3)	835 (27.3)	< 0.001
Inhospital hemodialysis, *n* (%)	250 (7.0)	193 (6.3)	< 0.001
Inhospital peritoneal_dialysis, *n* (%)	8 (0.2)	8 (0.3)	0.615
Inhospital CRRT, *n* (%)	587 (16.4)	449 (14.7)	< 0.001
Troponin_max (ng/mL)	0.2 [0.1, 1.2]	0.2 [0.1, 0.9]	< 0.001
Troponin_min (ng/mL)	0.1 [0.1, 0.5]	0.1 [0.0, 0.4]	< 0.001
Troponin_mean (ng/mL)	0.2 [0.1, 0.8]	0.2 [0.1, 0.7]	< 0.001
WBC_max (K/µL)	14.1 [10.5, 19.3]	13.6 [10.1, 18.4]	< 0.001
WBC_min (K/Ul)	6.8 [5.3, 8.6]	6.7 [5.2, 8.3]	< 0.001
WBC_mean (K/Ul)	10.0 [7.8, 12.7]	9.6 [7.6, 12.1]	< 0.001
RBC_max (m/µL)	3.7 [3.3, 4.2]	3.7 [3.3, 4.2]	0.029
RBC_min (m/Ul)	2.8 [2.4, 3.3]	2.8 [2.4, 3.2]	0.144
RBC_mean (m/Ul)	3.2 [2.9, 3.6]	3.2 [2.9, 3.6]	0.176
Hemoglobin_max (g/dL)	11.1 [9.9, 12.4]	11.1 [10.0, 12.4]	0.012
Hemoglobin_min (g/dL)	8.2 [7.2, 9.6]	8.2 [7.2, 9.6]	0.034
Hemoglobin_mean (g/dL)	9.6 [8.6, 10.7]	9.6 [8.7, 10.7]	0.049
Hematocrit_max (%)	34.1 [31.0, 38.1]	34.1 [31.0, 38.1]	0.473
Hematocrit_min (%)	25.3 [22.3, 29.7]	25.2 [22.3, 29.6]	0.949
Hematocrit_mean (%)	29.4 [26.9, 32.8]	29.4 [26.9, 32.7]	0.838
Platelet_max (K/µL)	247.0 [186.0, 325.0]	250.5 [191.0, 329.0]	< 0.001
Platelet_min (K/µL	136.0 [100.0, 185.0]	138.0 [102.0, 187.0]	< 0.001
Platelet_mean (K/µL)	186.2 [144.1, 239.8]	189.3 [148.0, 241.4]	< 0.001
ALT_max (IU/L)	27.0 [16.0, 62.0]	25.0 [16.0, 49.0]	< 0.001
ALT_min (IU/L)	18.0 [12.0, 31.0]	18.0 [12.0, 29.0]	< 0.001
ALT_mean (IU/L)	23.2 [15.0, 45.5]	21.8 [14.0, 39.0]	< 0.001
AST_max (IU/L)	42.0 [24.0, 104.0]	38.0 [23.0, 82.0]	< 0.001
AST_min (IU/L)	25.0 [18.0, 38.0]	24.0 [17.0, 35.0]	< 0.001
AST_mean (IU/L)	33.3 [22.0, 63.0]	31.0 [21.0, 52.2]	< 0.001
ALP_max (IU/L)	93.0 [70.0, 134.0]	90.0 [68.0, 125.8]	< 0.001
ALP_min (IU/L)	77.0 [59.0, 102.0]	76.0 [58.0, 100.0]	< 0.001
ALP_mean (IU/L)	86.0 [67.0, 115.5]	84.0 [66.0, 111.0]	< 0.001
Bilirubin_total_max (mg/dL)	0.6 [0.4, 1.0]	0.6 [0.4, 0.9]	< 0.001
Bilirubin_total_min (mg/dL)	0.4 [0.3, 0.7]	0.4 [0.3, 0.7]	< 0.001
Bilirubin_total_mean (mg/dL)	0.5 [0.4, 0.8]	0.5 [0.3, 0.8]	< 0.001
Creatinine_max (mg/dL)	2.4 [1.6, 4.0]	2.2 [1.6, 3.7]	< 0.001
Creatinine_min (mg/dL)	1.4 [1.1, 2.0]	1.4 [1.1, 1.9]	< 0.001
Creatinine_mean (mg/dL)	1.8 [1.4, 2.9]	1.8 [1.3, 2.7]	< 0.001
BUN_max (mg/dL)	52.0 [36.0, 77.0]	50.0 [34.0, 73.0]	< 0.001
BUN_min (mg/dL)	24.0 [17.0, 36.0]	23.0 [17.0, 34.0]	< 0.001
BUN_mean (mg/dL)	38.4 [27.0, 54.0]	36.3 [26.1, 50.9]	< 0.001
Potassium_max (mEq/L)	5.0 [4.6, 5.6]	5.0 [4.6, 5.5]	< 0.001
Potassium_min (mEq/L)	3.6 [3.3, 4.0]	3.6 [3.4, 3.9]	0.278
Potassium_mean (mEq/L)	4.3 [4.0, 4.6]	4.3 [4.0, 4.5]	< 0.001
Sodium_max (mEq/L)	142.0 [140.0, 145.0]	142.0 [140.0, 145.0]	0.049
Sodium_min (mEq/L)	134.0 [131.0, 137.0]	135.0 [131.0, 137.0]	0.005
Sodium_mean (mEq/L)	138.3 [136.0, 140.7]	138.3 [136.2, 140.6]	0.386
Total_calcium_max (mg/dL)	9.1 [8.7, 9.6]	9.1 [8.7, 9.5]	0.533
Total_calcium_min (mg/dL)	8.0 [7.6, 8.4]	8.1 [7.7, 8.5]	< 0.001
Total_calcium_mean (mg/dL)	8.6 [8.2, 8.9]	8.6 [8.2, 8.9]	< 0.001
Free_calcium_max (mmol/L)	1.2 [1.1, 1.2]	1.2 [1.1, 1.2]	< 0.001
Free_calcium_min (mmol/L)	1.1 [1.0, 1.1]	1.1 [1.0, 1.1]	< 0.001
Free_calcium_mean (mmol/L)	1.1 [1.1, 1.2]	1.1 [1.1, 1.2]	< 0.001
Magnesium_max (mg/dL)	2.5 [2.3, 2.8]	2.5 [2.3, 2.8]	0.234
Magnesium_min (mg/dL)	1.8 [1.7, 2.0]	1.8 [1.7, 2.0]	0.559
Magnesium_mean (mg/dL)	2.1 [2.0, 2.3]	2.1 [2.0, 2.3]	0.002
Phosphate_max (mg/dL)	4.9 [4.0, 6.2]	4.7 [4.0, 5.8]	< 0.001
Phosphate_min (mg/dL)	2.8 [2.2, 3.4]	2.8 [2.3, 3.3]	< 0.001
Phosphate_mean (mg/dL)	3.8 [3.2, 4.5]	3.7 [3.2, 4.3]	< 0.001
INR_max	1.5 [1.2, 2.3]	1.4 [1.2, 2.0]	< 0.001
INR_min	1.1 [1.0, 1.2]	1.1 [1.0, 1.2]	< 0.001
INR_mean	1.3 [1.1, 1.6]	1.2 [1.1, 1.5]	< 0.001
PT_max (s)	16.1 [13.6, 24.3]	15.7 [13.4, 22.3]	< 0.001
PT_min (s)	12.4 [11.4, 13.8]	12.2 [11.4, 13.4]	< 0.001
PT_mean (s)	14.1 [12.6, 17.2]	13.8 [12.5, 16.1]	< 0.001
PTT_max (s)	45.4 [31.9, 105.2]	42.5 [31.4, 97.8]	< 0.001
PTT_min (s)	27.6 [25.3, 30.7]	27.4 [25.2, 30.2]	< 0.001
PTT_mean (s)	35.9 [29.4, 54.2]	34.7 [29.1, 52.0]	< 0.001
Glucose_max (mg/dL)	194.0 [149.0, 271.0]	189.0 [147.0, 261.0]	< 0.001
Glucose_min (mg/dL)	88.0 [74.0, 103.0]	87.0 [74.0, 101.0]	< 0.001
Glucose_mean (mg/dL)	131.2 [111.8, 162.7]	128.2 [110.6, 157.7]	< 0.001
SOFA	6.0 [4.0, 8.0]	5.0 [3.0, 7.0]	< 0.001
BMI (kg/m^2^)	28.0 [24.2, 32.6]	28.1 [24.3, 32.6]	0.018
sbp_max (mmHg)	155.0 [140.0, 172.0]	156.0 [141.0, 173.0]	< 0.001
sbp_min (mmHg)	85.0 [75.0, 96.0]	87.0 [78.0, 97.0]	< 0.001
sbp_mean (mmHg)	117.7 [107.9, 129.5]	119.1 [109.9, 130.6]	< 0.001
dbp_max (mmHg)	93.0 [80.0, 109.0]	93.0 [80.0, 109.0]	0.08
dbp_min (mmHg)	39.0 [33.0, 46.0]	40.0 [34.0, 47.0]	< 0.001
dbp_mean (mmHg)	58.7 [52.8, 65.2]	59.0 [53.2, 65.7]	< 0.001
mbp_max (mmHg)	108.0 [96.0, 125.0]	108.0 [96.0, 124.0]	0.52
mbp_min (mmHg)	53.0 [46.0, 60.0]	54.0 [47.0, 61.0]	< 0.001
bmp_mean (mmHg)	74.9 [69.4, 81.4]	75.6 [70.1, 82.0]	< 0.001
HR_max (beats/min)	102.0 [88.0, 120.0]	100.0 [88.0, 116.0]	< 0.001
HR_min (beats/min)	62.0 [55.0, 70.0]	62.0 [56.0, 70.0]	< 0.001
HR_mean (beats/min)	79.8 [71.4, 88.9]	79.0 [71.0, 87.4]	< 0.001
spo2_max	100[100.0, 100.0]	100[100.0, 100.0]	0.004
spo2_min	90.0 [86.0, 93.0]	91.0 [88.0, 93.0]	< 0.001
spo2_mean	96.7 [95.6, 97.8]	96.7 [95.6, 97.8]	0.005

*los_icu* length of stay in intensive care unit, *scr* serum creatinine, *eGFR* estimated glomerular filtration rate, *CKD* chronic kidney disease, *ACS* acute coronary syndrome, *HT* hypertension, *PCI* percutaneous coronary intervention, *CABG* coronary artery bypass grafting, *NOAC* non-vitamin K Antagonist Oral Anticoagulant, *CRRT* continuous renal replacement therapy, *max* maximum, *min* minimum, *WBC* white blood cell, *RBC* red blood cell, *ALT* alanine aminotransferase, *AST* aspartate aminotransferase, *ALP* alkaline phosphatase, *BUN* blood urea nitrogen, *INR* International Normalized Ratio, *PT* prothrombin time, *PTT* partial thromboplastin time, *SOFA* sequential organ failure assessment, *sbp* systolic blood pressure, *dbp* diastolic blood pressure, *mbp* mean blood pressure, *HR* heart rate, *spo2* oxyhemoglobin saturation

**Table 2:** The performance of different machine learning models

Machine learning	AUC	Precision
Random forest	0.9	0.696
Logistic regression	0.921	0.754
SVM	0.937	0.773
Decision tree	0.721	0.323
GBDT	0.946	0.778
KNN	0.747	0.385
NN	0.9	0.601
XGBOOST	0.939	0.776

### Feature selection

**Fig. 2:**
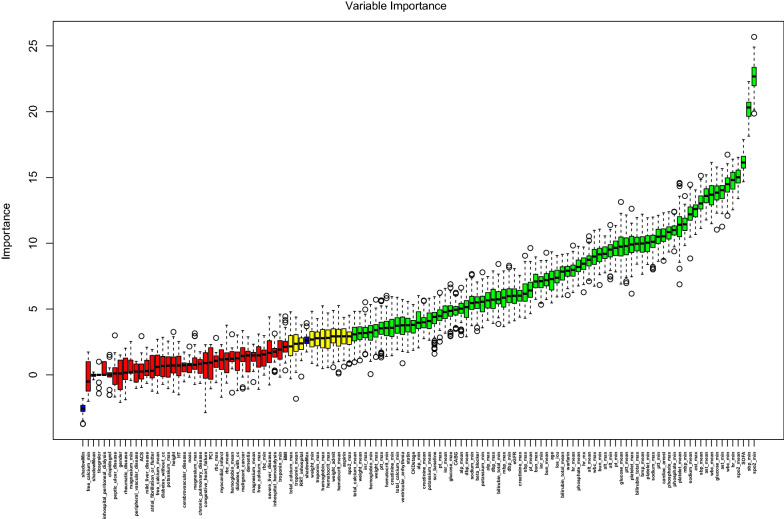
Feature selection analyzed by Boruta algorithm. The horizontal axis is the name of each variable, and the vertical axis is the *Z*-value of each variable. The box plot shows the *Z*-value of each variable in the model calculation. The green boxes represent the 76 important variables, the yellow represents tentative attributes, and the red represents unimportant variables. *los_icu* length of stay in intensive care unit, *scr* serum creatinine, *eGFR* estimated glomerular filtration rate, *CKD* chronic kidney disease, *ACS* acute coronary syndrome, *HT *hypertension, *PCI* percutaneous coronary intervention, *CABG* coronary artery bypass grafting, *NOAC* Non-vitamin K Antagonist Oral Anticoagulant, *CRRT* continuous renal replacement therapy, *max* maximum, *min* minimum, *WBC* white blood cell, *RBC* red blood cell, *ALT* alanine aminotransferase, *AST* aspartate aminotransferase, *ALP* alkaline phosphatase, *BUN* blood urea nitrogen, *INR* International Normalized Ratio, *PT* prothrombin time, *PTT* partial thromboplastin time, *SOFA* sequential organ failure assessment, *sbp* systolic blood pressure, *dbp* diastolic blood pressure, *mbp* mean blood pressure, *HR* heart rate, *spo2* oxyhemoglobin saturation

According to the Boruta algorithm analysis, 76 of 124 variables most closely associated with in-hospital mortality were selected (Fig. 2). Based on the *Z*-values, the top twenty variables are the history of cardiac arrest, sequential organ failure assessment (SOFA) score, the maximum values of aspartate aminotransferase (AST) and phosphate, the average values of spo2, white blood cell (WBC), AST, systolic blood pressure (sbp), sodium and platelet, and the minimum values of oxyhemoglobin saturation (spo2), SBP, heart rate, WBC, AST, glucose, phosphate, partial thromboplastin time (PTT), and mean blood pressure (mbp). Although the *Z*-values for acute coronary syndromes and diabetes were lower than the maximum *Z*-value of shadow feature, they were included in the analyses based on clinical experience. Therefore, a total of 78 variables were selected for the machine learning model development process.

### Machine learning model development and comparisons

**Fig. 3:**
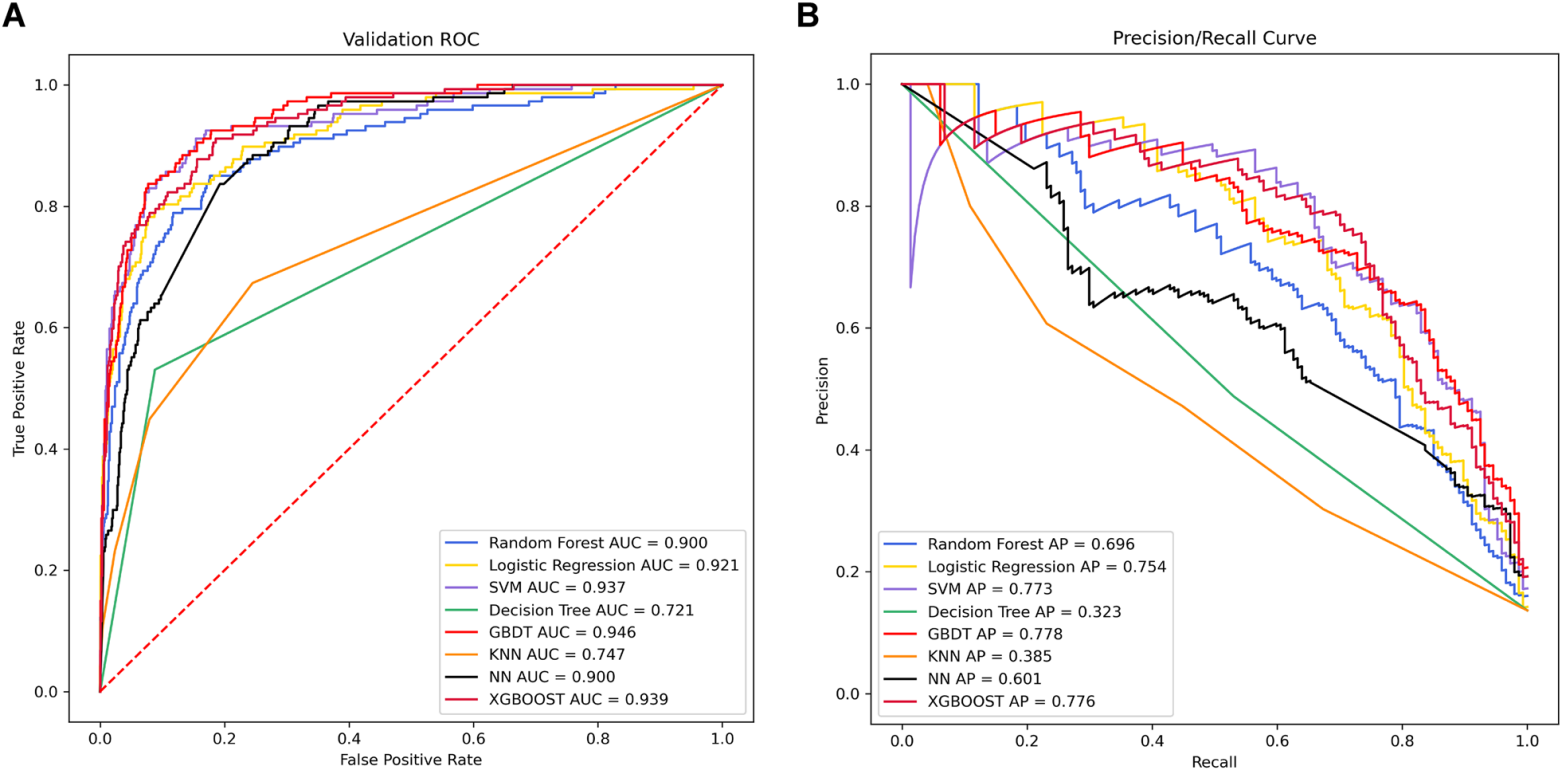
Discrimination performance of eight machine learning models. **A** ROC of eight machine learning models. **B** P-R curves of eight machine learning models. The GBDT algorism exhibited the best performance both in ROC and P-R curves. *ROC* Receiver Operating Characteristic, *P-R curve* precision/recall curve, *SVM* support vector machine, *GBDT* Gradient Boosting Decision Tree Machine, *KNN* k-nearest neighbors, *NN* neural network, *XGBoost* Extreme Gradient Boosting, *AUC* area under the curve

Eight machine learning models were generated to predict the in-hospital mortality in CKD patients with CAD. Among the eight models, GBDT had the best predictive value of in-hospital death, with AUC = 0.946 and AP = 0.778. Figure 3 exhibited the discrimination performance of these machine learning models via ROC and P-R curves after ten cross-fold-validation in the test set. The SVM (AUC = 0.937), XGBOOST (AUC = 0.939), and GBDT had superior performance in the predictive ability for in-hospital death of CKD patients with CAD compared to the traditional logistic regression model. A set of detailed performance metrics for various machine learning models is presented in Table 2.

### Visualization by SHAP

**Fig. 4:**
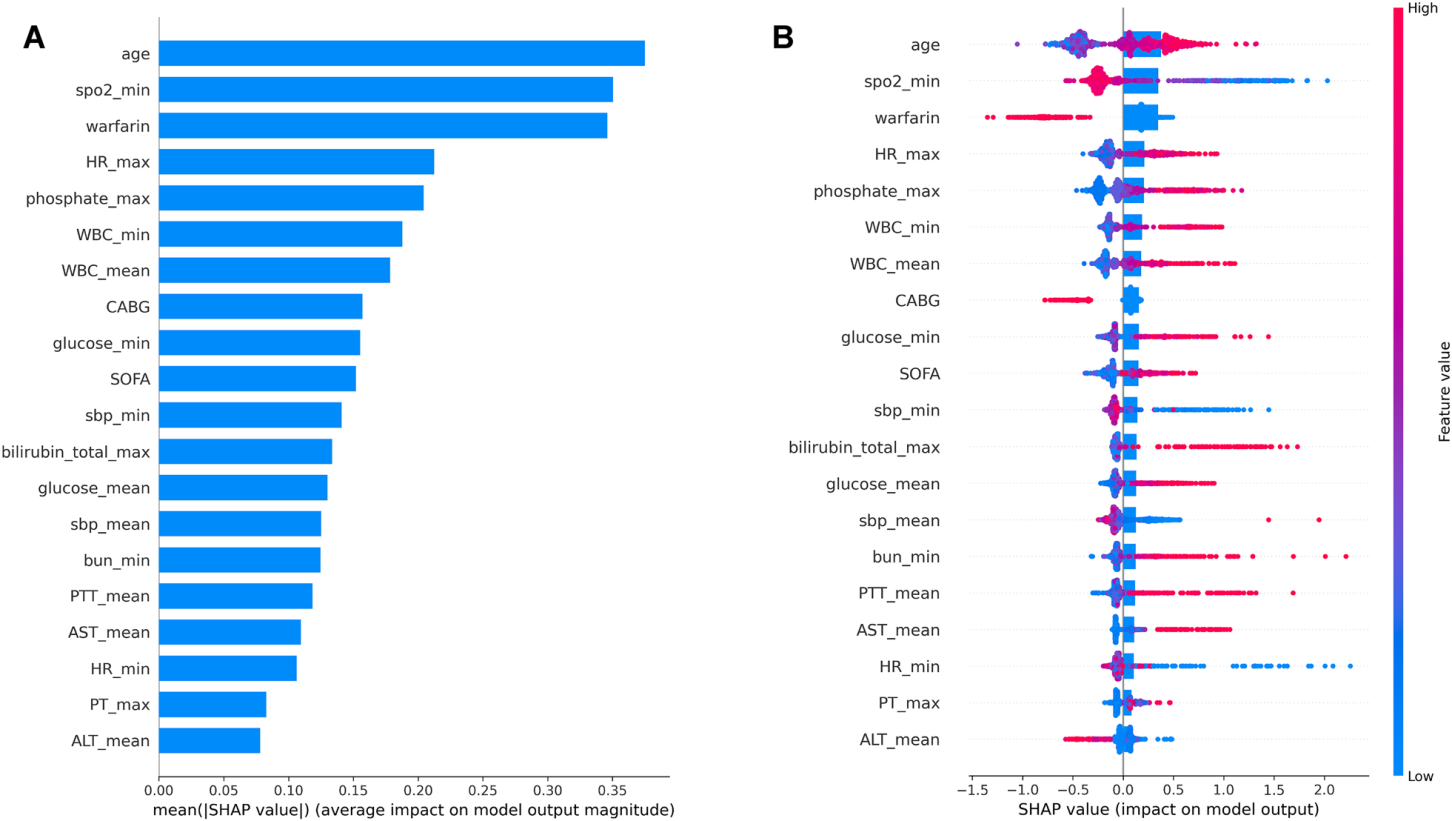
SHAP analysis result. **A** Bar charts that rank the importance of the top 20 significant variables most correlated to in-hospital death in GBDT model. **B** Impact of each feature on the in-hospital mortality in GBDT model by SHAP values. *GBDT* Gradient Boosting Decision Tree Machine, *SHAP* Shapley Additive Explanations, *spo2* oxyhemoglobin saturation, *HR* heart rate, *WBC* white blood cell, *CABG* coronary artery bypass grafting, *SOFA* sequential organ failure assessment, *sbp* systolic blood pressure, *BUN* blood urea nitrogen, *PTT* partial thromboplastin time, *ALT* alanine aminotransferase, *AST* aspartate aminotransferase, *PT* prothrombin time

The SHAP algorithm was conducted to visually exhibit each factor's importance to the hospital mortality predicted by the GBDT model. Figure 4A shows the feature importance plot, including 20 significant variables most correlated to in-hospital death in descending order. The age factor had the most potent predictive power, followed by the minimum value of spo2 and warfarin. Figure 4B presents whether that feature is high (in red) or low (in blue) for that observation according to the SHAP value. The utilization of warfarin has a negative impact on in-hospital mortality.

### Subgroup analysis

Subgroup analyses were conducted stratifying by ACS and dialysis condition. Age was no longer the most potent predictive factor in ACS and non-ACS patients and warfarin dropped out of the top 20 significant variables in ACS patients. SOFA score had the most potent predictive value in dialysis patients followed by glucose level. Interestingly, phosphate level was one of the top 20 influencing factors in non-dialysis patients, but its predictive value in dialysis patients was limited (Additional file 1: Fig. S1, Additional file 2: Fig. S2).

### External validation

**Fig. 5:**
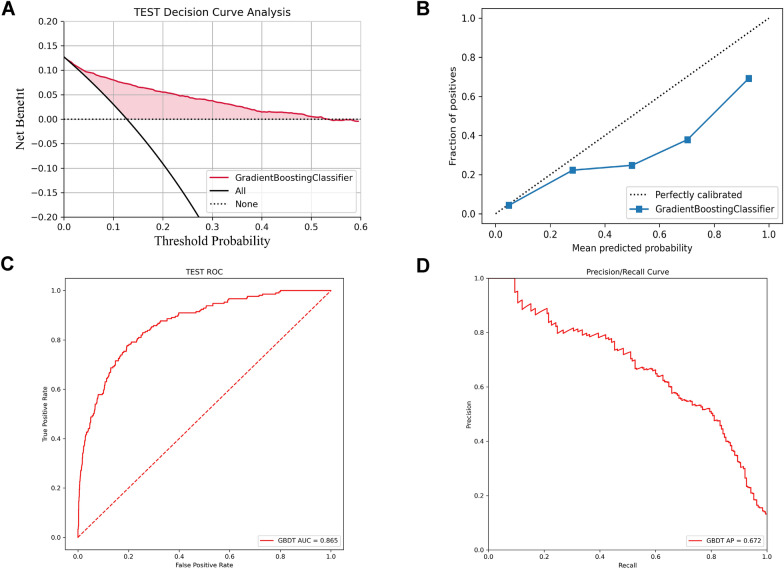
External validation for the GBDT model in the eICU-CRD dataset. **A** DCA curve of the GBDT model in external validation. **B** calibration curve of the GBDT model in external validation. **C** ROC of the GBDT model in external validation. **D** P-R curves of the GBDT models in external validation. DCA showed the GBDT model had some net benefit compared with the “treat-none” or “treat-all” strategies with a certain degree of clinical utility. The AUC (0.865) and AP (0.672) results demonstrated the GBDT model had good predictive values in external validation. *DCA* decision curve analysis, *ROC* Receiver Operating Characteristic, *P-R curve* precision/recall curve, *GBDT* Gradient Boosting Decision Tree Machine, *eICU-CRD* Telehealth Intensive Care Unit Collaborative Research Database

A total of 1657 CKD patients with CAD were extracted from the eICU-CRD database as an external validation dataset to verify the predictive accuracy of the selected GBDT model. Additional file 3: Table S1 exhibits the baseline characteristics of these patients. A total of 211 (12.7%) patients died during hospitalization. Taken together, GBDT had good predictive values (AUC = 0.865, AP = 0.672), while the clinical value was limited in the validation cohort based on the result of DCA and calibration curve (Fig. 5).

## Discussion

Patients with CKD and CAD became more and more popular in recent decades. And mortality in patients suffering from these two conditions is twice as compared to patients with CAD alone [4]. Despite the increased incidence and incredibly lethal, their patients were excluded from most clinical trials due to the disease complexity and treatment conflicts. To date, factors associated with the prognosis in CKD patients with CAD were not clear and current risk stratification tools could not be applied to these patients. With the development of artificial intelligence, accurate prediction of these complex conditions could be achieved using machine learning methods.

MIMIC-IV and eICU-CRD were large-scale and high-quality databases performed in many crucial pieces of research in recent years. In this retrospective study, CKD patients with CAD admitted to ICU were extracted from MIMIC-IV to develop predictive models for in-hospital mortality via various ML algorithms. The GBDT model outperformed the predictive performance of seven other ML algorithms, including LR, RF, Decision Tree, KNN, SVM, NN, and XGBoost, according to the features selected by the Boruta algorithm. Next, the SHAP method was conducted to explain GBDT visually, ensuring clinical interpretability and facilitating the utilization of the prediction model. The performance and clinical application value of GBDT were also validated by an external set from the eICU-CRD database. This is the first prediction method especially for CKD patients with CAD to evaluate the in-hospital mortality with precise efficiency in two large cohorts, which means good generalization to extend to clinical practice.

Depending on the visualization technique SHAP, our study identified several crucial variables related to the in-hospital mortality of patients with CKD and CAD in the ICU. This study identified a factor strongly associated with the in-hospital mortality observed in our study which was serum phosphate. Previous studies have shown that elevated serum inorganic phosphorous (P) is tightly associated with cardiac death in CKD patients [15]. A national study illustrated that hyperphosphatemia could lead to a predisposition to metastatic calcification and the development and progression of secondary hyperparathyroidism, which may contribute to the abundant morbidity and mortality of patients with ESRD [16]. Another research with a 2-year follow-up also identified strong relationships between hyperphosphatemia and cardiac causes of death in hemodialysis patients [17]. Moreover, a cross-sectional study showed elevated serum levels of P were significantly related to calcified coronary atherosclerotic plaque detected by cardiac computed tomography, even in patients with normal kidney function [18]. The previous studies exhibited the significance of P in prognosis in CKD patients. In our study, we focused on CKD patients with CAD and showed that serum P was a strong predictor of in-hospital mortality. Therefore, phosphate is a promising therapeutic target to improve the clinical outcome in CKD patients with CAD. Both dietary and pharmacological therapeutic strategies should be used to reduce of serum phosphate levels to prevent hyperphosphatemia in CKD patients with CAD.

Whether Coronary Artery Bypass Grafting (CABG) or PCI is the better approach for revascularization of CAD in CKD patients was still controversial. Several observational studies reported CABG was associated with lower mortality than PCI in CKD patients [19–21]. But the Coronary REvascularization Demonstrating Outcome Study in Kyoto PCI/CABG Registry Cohort-2 study showed the risk of all-cause death was similar between PCI and CABG in ESRD patients requiring dialysis [22], which was consistence with the result of ISCHEMIA-CKD research [3]. Another meta-analysis also pointed out that patients with stage 3–5 CKD who underwent either approach to revascularization did not experience significant differences in mortality. However, CABG significantly reduced the myocardial infarction risks and required fewer additional revascularization procedures [23]. Different results in these studies might be attributed to different study participants, some focused on advanced CKD patients, while others focused on ESRD patients. Our study included patients with all staged CKD, ML visible results showed that both PCI and CABG were beneficial to the prognosis of CKD patients with CAD, and CABG was a more critical feature than PCI to the in-hospital mortality in those patients in ICU.

A growing number of machine learning applications in cardiovascular medicine have been made possible by the development of artificial intelligence [24, 25]. Using machine learning, it has been possible to predict death risk among CAD patients more accurately than before. Motwani et al. constructed a boosted ensemble algorithm combining clinical and coronary computed tomographic angiography (CCTA) to predict 5-year all-cause mortality with higher AUC (0.79) than clinical or CCTA metrics alone [26]. Silva et al. established a prognostic model using health conditions, including age and maximal exercise capacity, to precisely predict the mortality of CAD patients via the survival tree (ST) algorithm (C-index 0.729) [27]. In addition, Pezel and colleagues developed multiple fractional polynomial algorithm ML models, including 31,752 consecutive patients, to predict 10-year death [28]. This ML model also has a higher prognostic value than traditional clinical or Cardiac Magnetic Resonance scores (AUC 0.76). However, the mechanism of CKD combined with CAD is more complex and harder to explain than the mechanism of CAD alone [4]. For example, statin lipid-lowering therapy is still contradictory in improving the prognosis of patients with ESRD and CAD [29]. Predictions based on the traditional model cannot be made with reasonable accuracy and comprehensiveness for patients suffering from such complex diseases [5, 30]. For this reason, machine learning is of great significance.

The GBDT algorithm, also known as the multiple additive regression trees, has more accurate predictive ability and sophisticated algorithms than the LR, decision tree, and random forest algorithms [31]. It has many nonlinear transformations and solid, expressive ability, and does not require complex feature engineering and transformation [32]. The XGBoost model, a modified GBDT algorithm, could cope efficiently and flexibly with missing data and combines weak predictors to produce accurate predictions [33]. The no free lunch theorem (NFL) illustrates that the expected performance of each learning algorithm is the same if all possible problems are considered, which means there is no single, universal best machine learning algorithm for every situation [34]. Among eight ML models, the GBDT model performed the best clinical predictive value in in-hospital mortality risks in this kind of patient.

The advantages of this study were that it was the first study focusing on the in-hospital mortality for CKD patients with CAD in ICU based on a public database and constructed an ML model to predict it with external validation. Some limitations must be acknowledged. First, MIMIC-IV was a single-center database; most white patients may lead to racial bias and limit the applicability to other populations. However, external validation was applied using data from a multicenter database, eICU-CRD. Second, the deviation of missing data was inevitable because the data were extracted from the open public database. We performed fully conditional specification (FCS) implemented by the MICE algorithm to multiply and impute the missing data. Third, the selection bias was inevitable because this was a retrospective and observative study. Data were extracted from two different databases as internal and external sets, and further multicenter and large-scale clinical research was still needed. Nevertheless, the constructed ML model still may contribute to clinicians improving the prognosis and treating CKD patients with CAD at high risk in ICU timely. Collecting clinical data on ICU patients have been difficult due to the impact of the *CoronaVirusDisease2019* outbreak. Public databases have helped tide clinical workers over worldwide. But more prospective multicenter clinical studies should also be established for further research.

## Conclusions

In conclusion, machine learning algorithms can be reliable tools for accurately predicting the in-hospital mortality risk for CKD patients with CAD in the ICU. GBDT technology had the best predictive performance, which may provide optimal resource allocation and reduce in-hospital mortality by tailoring precise management and implementing early interventions.

## Supplementary Information

**Additional file 1: Fig. S1** Subgroup analysis showed via SHAP plot stratified by ACS. A: Impact of each feature on the in-hospital mortality in non-ACS patients; B: Impact of each feature on the in-hospital mortality in ACS patients.

**Additional file 2: Fig. S2** Subgroup analysis showed via SHAP plot stratified by dialysis. A: Impact of each feature on the in-hospital mortality in non-dialysis patients; B: Impact of each feature on the in-hospital mortality in dialysis patients.

**Additional file 3: Table S1** Baseline characteristics of CKD patients with CAD in the external validation set (eICU-CRD).

## Data Availability

The data supporting this study’s findings are available from the Medical Information Mart for Intensive Care IV (MIMIC-IV), but restrictions apply to the availability of these data, which were used under license for the current study, and so are not publicly available. Data are, however, available from the author Shuoyan An (anshuoyan@126.com) upon reasonable request and with permission of MIMIC.

## Abbreviations

CKD: Chronic kidney disease
CAD: Coronary artery disease
MIMIC-IV: Medical Information Mart for Intensive Care IV
LR: Logistic regression
RF: Random forest
GBDT: Gradient Boosting Decision Tree Machine
KNN: K-nearest neighbors
SVM: Support Vector Machine
NN: Neural Network
XGBoost: Extreme Gradient Boosting
AP: Average precision
AUC: Area under the receiver operating characteristic curve
SHAP: Shapley Additive Explanations
eICU-CRD: Telehealth Intensive Care Unit Collaborative Research Database
ESRD: End-stage renal disease
ML: Machine learning
ICD-9: International Classification of Diseases and Ninth Revision
ICU: Intensive care unit
AP: Average precision
P-R curves: Precision/recall curves
DCA: Decision curve analysis
CCTA: Coronary computed tomographic angiography
NFL: No free lunch theorem
FCS: Fully conditional specification
los_icu: Length of stay in intensive care unit
scr: Serum creatinine
eGFR: Estimated glomerular filtration rate
ACS: Acute coronary syndrome
HT: Hypertension
PCI: Percutaneous coronary intervention
CABG: Coronary artery bypass grafting
NOAC: Non-vitamin K Antagonist Oral Anticoagulant
CRRT: Continuous renal replacement therapy
max: Maximum
min: Minimum
WBC: White blood cell
RBC: Red blood cell
ALT: Alanine aminotransferase
AST: Aspartate aminotransferase
ALP: Alkaline phosphatase
BUN: Blood urea nitrogen
INR: International Normalized Ratio
PT: Prothrombin time
PTT: Partial thromboplastin time
SOFA: Sequential organ failure assessment
sbp: Systolic blood pressure
dbp: Diastolic blood pressure
mbp: Mean blood pressure
HR: Heart rate
spo2: Oxyhemoglobin saturation
