# Supplementary figures and images for: The prediction of in-hospital mortality in chronic kidney disease patients with coronary artery disease using machine learning models
Source: Eur J Med Res. 2023 Jan 18;28:33. doi: 10.1186/s40001-023-00995-x (PMC9847092; doi:10.1186/s40001-023-00995-x)

**A**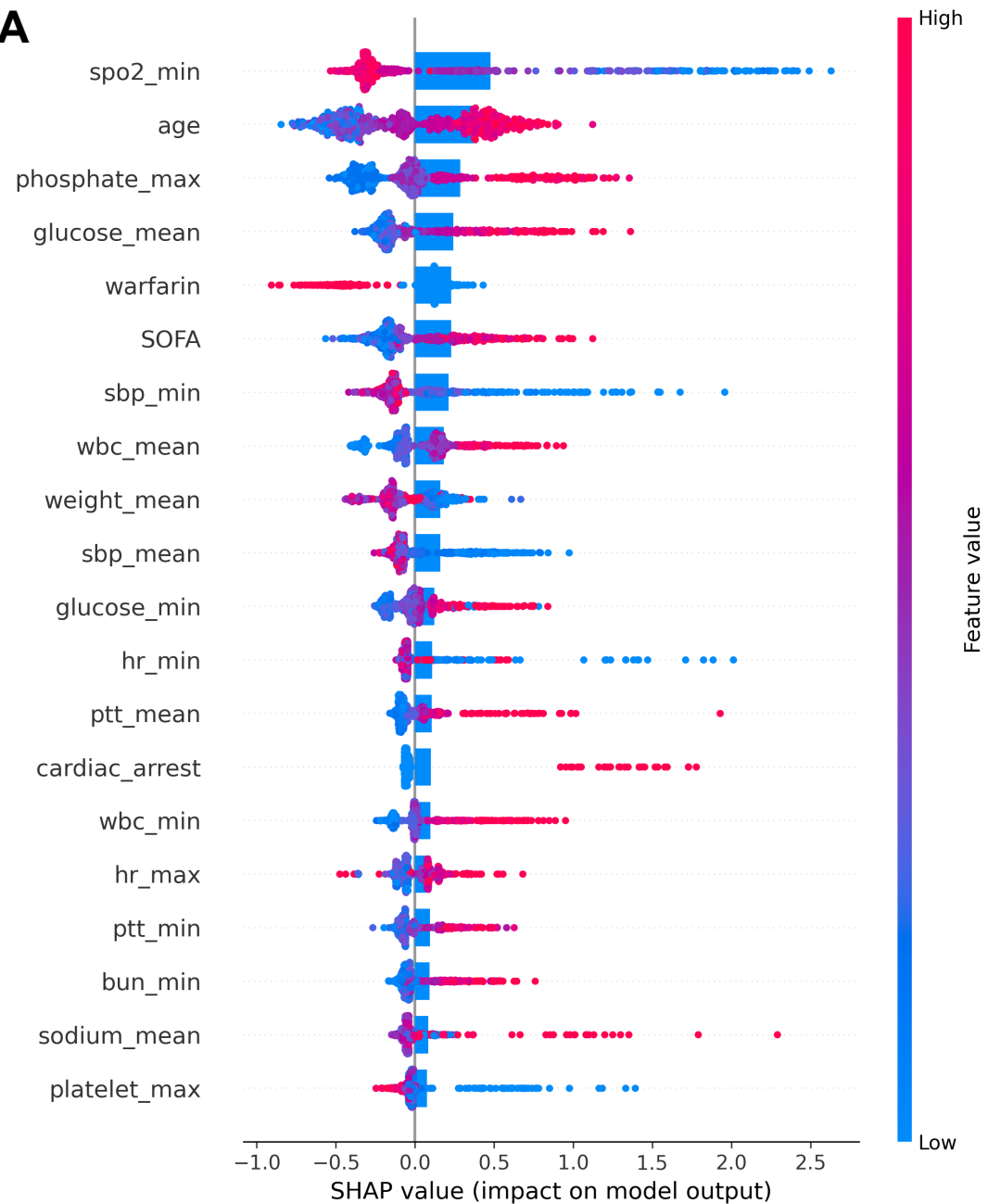**B**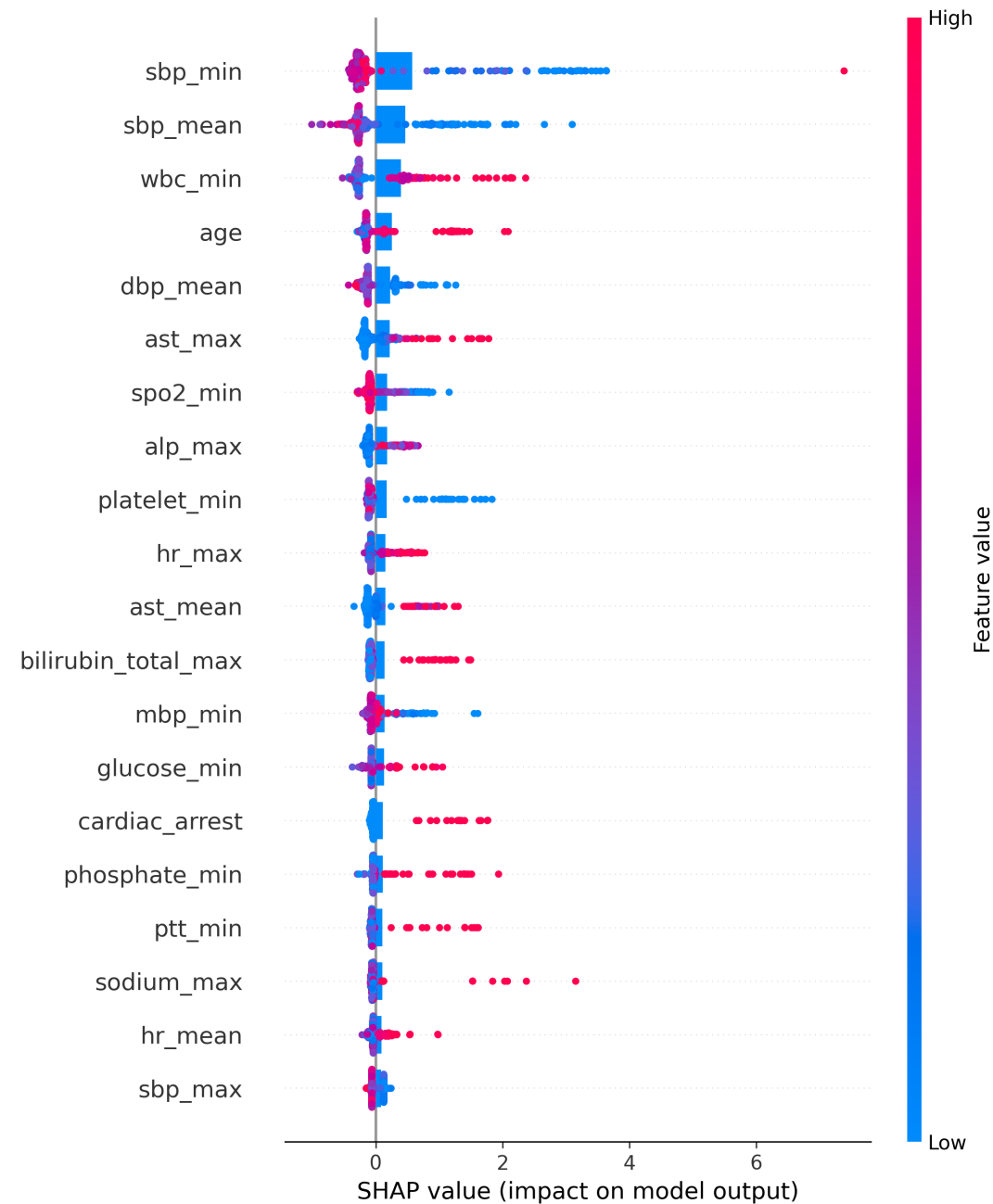

Supplement: Supplementary file 1 — Additional file 1: Fig. S1 Subgroup analysis showed via SHAP plot stratified by ACS. A: Impact of each feature on the in-hospital mortality in non-ACS patients; B: Impact of each feature on the in-hospital mortality in ACS patients. [file 40001_2023_995_MOESM1_ESM.pdf]

**A**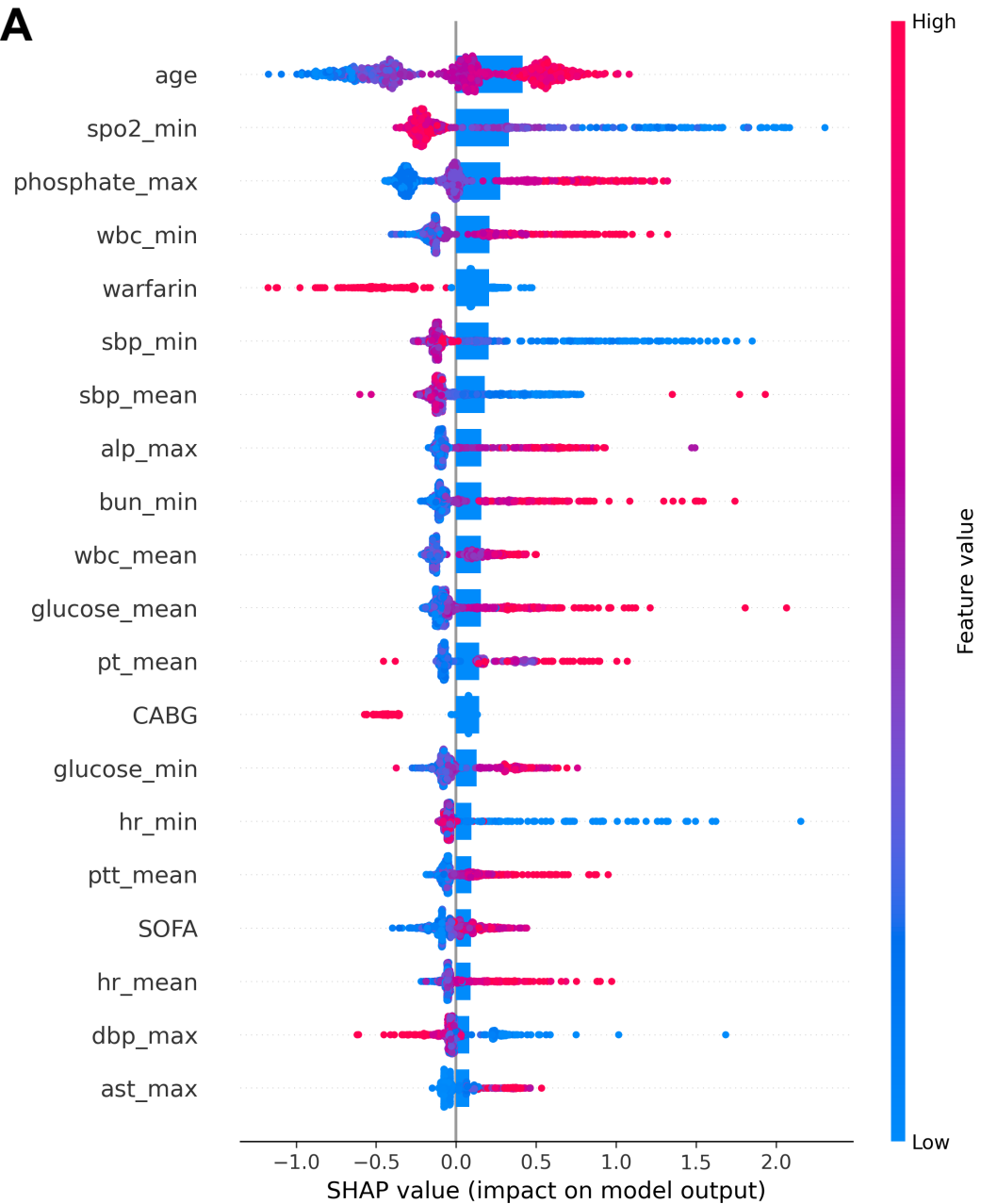**B**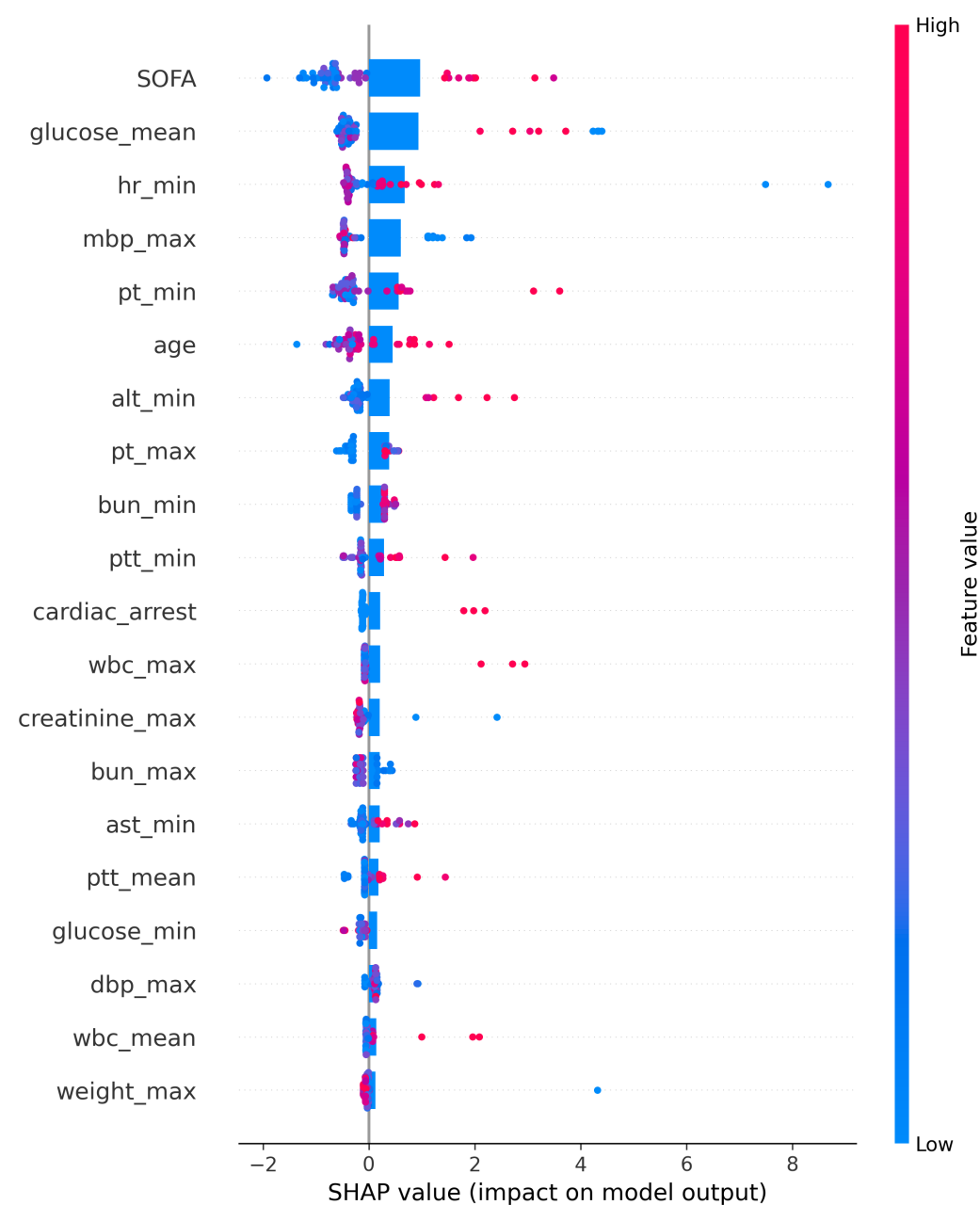

Supplement: Supplementary file 2 — Additional file 2: Fig. S2 Subgroup analysis showed via SHAP plot stratified by dialysis. A: Impact of each feature on the in-hospital mortality in non-dialysis patients; B: Impact of each feature on the in-hospital mortality in dialysis patients. [file 40001_2023_995_MOESM2_ESM.pdf]
